# Using Super-Imposition by Translation And Rotation (SITAR) to relate pubertal growth to bone health in later life: the Medical Research Council (MRC) National Survey of Health and Development

**DOI:** 10.1093/ije/dyw134

**Published:** 2016-07-27

**Authors:** TJ Cole, D Kuh, W Johnson, KA Ward, LD Howe, JE Adams, R Hardy, KK Ong

**Affiliations:** 1 Population, Policy and Practice Programme, UCL Institute of Child Health, London, UK; 2 MRC Unit for Lifelong Health and Ageing at UCL, London, UK; 3 School of Sport, Exercise and Health Sciences, Loughborough University, UK; 4 MRC Human Nutrition Research, Cambridge, UK; 5 MRC Integrative Epidemiology Unit, School of Social and Community Medicine, University of Bristol, Bristol, UK; 6 Clinical Radiology and Academic Health Science Centre, Manchester Royal Infirmary & University, Manchester, UK and; 7 MRC Epidemiology Unit, Institute of Metabolic Science, University of Cambridge, Cambridge, UK

**Keywords:** SITAR, ageing, height, weight, puberty, bone, density

## Abstract

**Background:** To explore associations between pubertal growth and later bone health in a cohort with infrequent measurements, using another cohort with more frequent measurements to support the modelling, data from the Medical Research Council (MRC) National Survey of Health and Development (2–26 years, 4901/30 004 subjects/measurements) and the Avon Longitudinal Study of Parents And Children (ALSPAC) (5–20 years) (10 896/74 120) were related to National Survey of Health and Development (NSHD) bone health outcomes at 60–64 years.

**Methods:** NSHD data were analysed using Super-Imposition by Translation And Rotation (SITAR) growth curve analysis, either alone or jointly with ALSPAC data. Improved estimation of pubertal growth parameters of size, tempo and velocity was assessed by changes in model fit and correlations with contemporary measures of pubertal timing. Bone outcomes of radius [trabecular volumetric bone mineral density (vBMD) and diaphysis cross-sectional area (CSA)] were regressed on the SITAR parameters, adjusted for current body size.

**Results:** The NSHD SITAR parameters were better estimated in conjunction with ALSPAC, i.e. more strongly correlated with pubertal timing. Trabecular vBMD was associated with early height tempo, whereas diaphysis CSA was related to weight size, early tempo and slow velocity, the bone outcomes being around 15% higher for the better vs worse growth pattern.

**Conclusions:** By pooling NSHD and ALSPAC data, SITAR more accurately summarized pubertal growth and weight gain in NSHD, and in turn demonstrated notable associations between pubertal timing and later bone outcomes. These associations give insight into the importance of the pubertal period for future skeletal health and osteoporosis risk.

**Key Messages**

- Super-Imposition by Translation And Rotation (SITAR) growth curve analysis is effective at summarizing pubertal growth.
- For cohorts with infrequent measurements, modelling jointly with a second cohort can improve the fit.
- Bone health at age 60–64 years is strongly associated with greater size, earlier timing and slower growth in puberty.

## Introduction

Puberty is a time of dramatic change in body size and composition, driven by a complex hormonal cascade that results in rapid weight gain and height gain, the timing of which varies widely between individuals.^1^ Studies using questionnaire-derived data on recalled age at menarche, a discrete event during late puberty in girls, indicate that the timing of puberty is influenced by both genetic^2^ and non-genetic factors, and that it has important relevance to later health outcomes.^3^ However, the regulation of pubertal changes in weight and height, and in particular the relevance of these growth traits to later outcomes, is difficult to study, for two reasons: the need for serial growth measurements over much of the second decade of life, and the need for a suitable methodology to model and summarize the pattern of pubertal growth to relate it to later outcome.

An example of this arises in our recent report on the associations between childhood growth and bone outcomes at 60–64 years^4^ in the Medical Research Council (MRC) National Survey of Health and Development (NSHD), a birth cohort born in one week in March 1946 and followed up over the subsequent 70 years.^5^^,^^6^ We found that greater height and weight growth during ‘pre-puberty’ and ‘post-puberty’ were positively associated with bone size, a predictor of fracture risk, at 60–64 years. Yet, given that 30–40% of skeletal mineral is accrued within two years of peak height velocity, it was surprising not to find stronger associations with growth during ‘puberty’. Whereas NSHD has more frequent growth measurements than many subsequent studies, the analysis was limited by the infrequent time-points around puberty (with just three sweeps at the ages of 11, 15 and 20 years) which were too sparse to easily test for associations with pubertal growth. Furthermore, the shape of the pubertal growth curve is complex, and summarizing it in a way that is suitable to relate to potential determinants or later outcomes is not straightforward.

In a life-course context, most studies have treated adolescent height and weight as exposures, either as single measurements (i.e. size) or as change over time (i.e. velocity), and few have considered the maturational clock (i.e. tempo, the timing of puberty) as an exposure, which may be confounded with size and velocity. Recently, the Super-Imposition by Translation And Rotation (SITAR) growth curve model has been shown to efficiently summarize pubertal growth based on these three parameters: size, velocity and tempo.^7–9^ Like most growth curve models, SITAR works best with frequent serial data, and this limits its use in cohort studies that have only sparse anthropometry. The purpose of this paper is to apply SITAR to NSHD growth data, and to explore whether the sparse nature of the data can be compensated for by augmenting them with data from another cohort of comparable size but with more frequent measurements—the Avon Longitudinal Study of Parents And Children (ALSPAC) cohort.^10^ To test whether the SITAR parameters, particularly tempo, were biologically meaningful, we related them to contemporaneously collected measures of pubertal timing in NSHD.

A companion paper relates the timing of puberty (reported menarche for girls and clinically assessed pubertal stage for boys) to later bone outcomes in NSHD and, in the same sample, compares these associations with the association with height tempo derived from our SITAR model.^11^ The current paper documents the process for augmenting NSHD with ALSPAC data in the SITAR analysis, and then tests the hypotheses that (i) this augmented model improves the correlations with reported pubertal status in NSHD and (ii) that the SITAR effects of size, velocity and tempo in NSHD in the full sample are associated with key bone health outcomes measured some 50 years later.^11^

## Methods

### NSHD

The NSHD is a socially stratified birth cohort of 2547 men and 2815 women of White European descent born during one week in March 1946 and followed with repeated data collections since then.^5^ Heights and weights were measured using standard protocols at ages 2, 4, 6, 7, 11 and 15 years, and self-reported at ages 20 and 26 years.

Pubertal timing was recorded at age 14–15 years. Mothers were asked their daughter's age at menarche (in months) and, where it had not yet occurred, it was imputed (*n* = 94) from reports of the subjects themselves who at 48 years were asked to recall their age at menarche.^12^ The school doctor assessed boys for genital development, voice breaking, pubic hair and axillary hair, leading to the four-point maturity scale prepubertal (1), early (2), advanced (3) and mature (4). For this analysis, the scale was reversed, so that a lower score corresponds to earlier puberty in both sexes.

At age 60–64 years, 1355 study members (658 men and 697 women) had a peripheral quantitative computed tomography (pQCT) scan of the radius.^4^ Among others, the following two bone outcome measures were derived: trabecular volumetric bone mineral density (vBMD, mg/cm^3^) at the distal 4% site, and diaphysis cross-sectional area (CSA, mm^2^) at the 50% site.

The study received Multi-Centre Research Ethics Committee approval, and informed consent was given by cohort participants.

### ALSPAC

The ALSPAC is a study of children born to 15 247 pregnant women living in Bristol with an expected delivery date between April 1991 and December 1992.^10^ Detailed information has been collected using self-administered questionnaires, data extraction from medical notes, and linkage to routine information systems and at research clinics.

Childhood weight and height were measured annually up to age 13 years, then at ages 15 and 17 years at dedicated ALSPAC Focus clinics by a trained research team. Parent-reported heights and weights were also included. Height was measured to the nearest 0.1 cm and weight to the nearest 0.1 kg. For the present analysis, data were restricted to the age range 5–20 years.

Ethical approval for the study was obtained from the ALSPAC Law and Ethics Committee and Local Research Ethics Committees.

### Data management

The data were examined for obvious outliers, and were further cleaned after preliminary fitting of the SITAR models by excluding points with standardized residuals exceeding 3 in absolute value.

### Data analysis

The analysis was performed in two stages. First, the height and weight data were analysed using SITAR growth curve analysis.^7^ This is a shape-invariant growth model such that all individuals are assumed to have the same underlying shape of growth curve, subject to three simple transformations. This mean curve is estimated along with three subject-specific parameters termed size, tempo and velocity that transform the mean curve to fit individual growth curves. The size parameter for each child shifts the fitted curve up/down, reflecting differences in size; the tempo parameter shifts it left/right, reflecting differences in puberty timing; and the velocity parameter stretches/shrinks the underlying age scale to make the curve shallower/steeper, reflecting differences in growth rate. The model fits the mean growth curve as a fixed effect natural cubic regression B-spline with specified degrees of freedom, and the parameters size, tempo and velocity are estimated as fixed effects and subject-specific random effects. The model was fitted with the nlme package^13^ and the first author's sitar package in the statistical language R.^14^ The SITAR formula is

(1) $$y_{it}=\alpha_{0}+\alpha_{i}+h\left(\frac{t-\beta_{0}-\beta_{i}}{e^{-\gamma_{0}-\gamma_{i}}}\right)+{ɛ}_{it},$$

where *y_it_* is the measurement for subject *i* at age *t*; *α_i_*, *β_i_* and γ*_i_* are, respectively, size, tempo and velocity random effects (along with corresponding fixed effects *α*_0_, *β*_0_ and γ_0_); *h*(.) is a cubic regression spline curve; and *ɛ_it_* are independent normally distributed errors.

Each model was fitted to the sexes separately. Initially, the models were fitted to the NSHD data alone (age 2–26 years), and then again with the ALSPAC data added (age 5–20 years). For the combined analyses, fixed effects were included in the model to distinguish between the two cohorts, allowing the cohort differences in mean size, tempo and velocity to be formally tested, leading to the extended formula

(2) $$y_{it}=\alpha_{0}+\alpha_{ALSPAC}+\alpha_{i}+h\left(\frac{t-\beta_{0}-\beta_{ALSPAC}-\beta_{i}}{e^{-\gamma_{0}-\gamma_{ALSPAC}-\gamma_{i}}}\right)+{ɛ}_{itNSHD}+{ɛ}_{itALSPAC},$$

where the cohort fixed effects have subscript ALSPAC. This assumes that the mean curves for NSHD and ALSPAC are the same after adjusting for the cohort fixed effects.

In addition, different residual variances were estimated for NSHD and ALSPAC, so that the reported residual variance refers to NSHD explicitly, whether or not ALSPAC data were in the model. The models were found to fit better after log-transforming age, and the resulting coefficients can be multiplied by 100 and viewed as percentage differences.^15^ Similarly, the standard deviations (SDs) of the tempo random effects are effectively coefficients of variation; the tempo random effects and SDs are here converted to units of years by multiplying by the geometric mean age.

The output from each SITAR analysis consisted of a set of subject-specific random effects for size, tempo and velocity, plus the corresponding fixed effects and (for the joint models) the cohort differences, along with fixed effects for the mean curve coefficients.

The second stage of the analysis involved just the NSHD subjects, with SITAR random effects from the joint models. Their two bone outcomes were each regressed in turn on the six SITAR random effects (three for height and three for weight), including sex as a main effect (this is termed Model 1). The outcomes were in addition adjusted for body size: height and weight at 60–64 years (Model 2). Models involving subsets of the six SITAR parameters were also fitted where the full results justified it, and sex interactions and quadratic terms were explored, though no clinically important effects were found. For all analyses, the bone outcomes and weight and height at age 60–64 years were log-transformed prior to analysis for allometric scaling purposes.

It is important to assess the potential impact of pubertal growth on outcome, analogous to attributable risk. In the simplest case, a model with a single SITAR parameter, this impact can be obtained directly by multiplying the regression coefficient by four times the parameter SD. This corresponds to the difference in outcome predicted for individuals with extreme growth patterns ±2 SDs from the mean for that parameter (i.e. comparing the third and ninety-seventh centiles). As the outcomes are logged, this difference can be multiplied by 100 and viewed as the percentage difference in outcome attributable to the contrast between the two growth patterns.^15^

However, it is less obvious how to measure impact when the model contains more than one SITAR parameter, as the parameters will be correlated, some of them highly, and the individual coefficients cannot be interpreted in isolation. To assess the impact of multiple SITAR parameters, the following method was used: the linear predictor corresponding to the SITAR parameters was derived as the sum of the parameters after multiplying each by its regression coefficient. This linear predictor shows how the predicted outcome varies across the spectrum of growth as represented by the combined SITAR parameters, adjusted for other covariates in the model, and the corresponding impact is summarized as four times the SD of the linear predictor. This is a multivariate extension of the simple case, and is the appropriate way to express the impact of the SITAR parameters as a single summary statistic.

## Results

### SITAR pubertal growth parameters in NSHD

**Figure 1.**
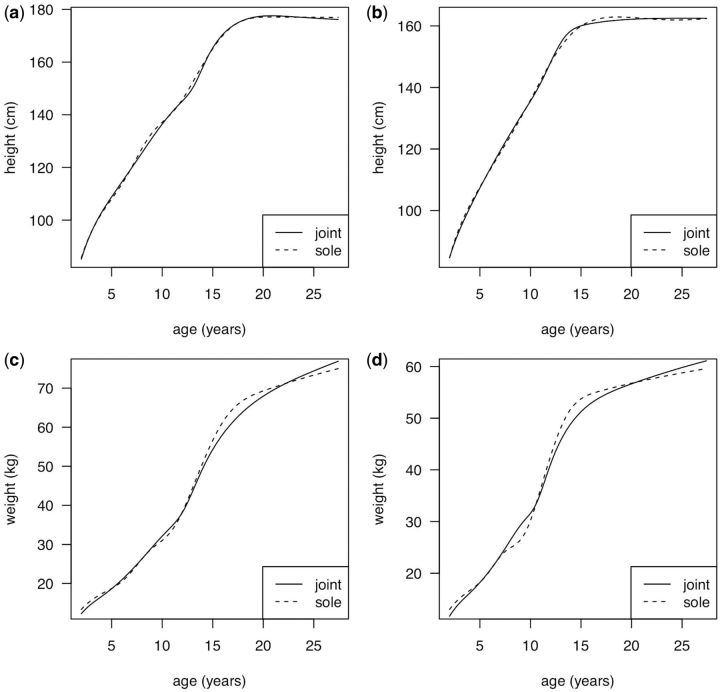
Mean NSHD growth curves as estimated by SITAR: height (above) and weight (below) in boys (left) and girls (right), estimated alone (dashed lines) or jointly with ALSPAC (solid lines).

**Figure 2.**
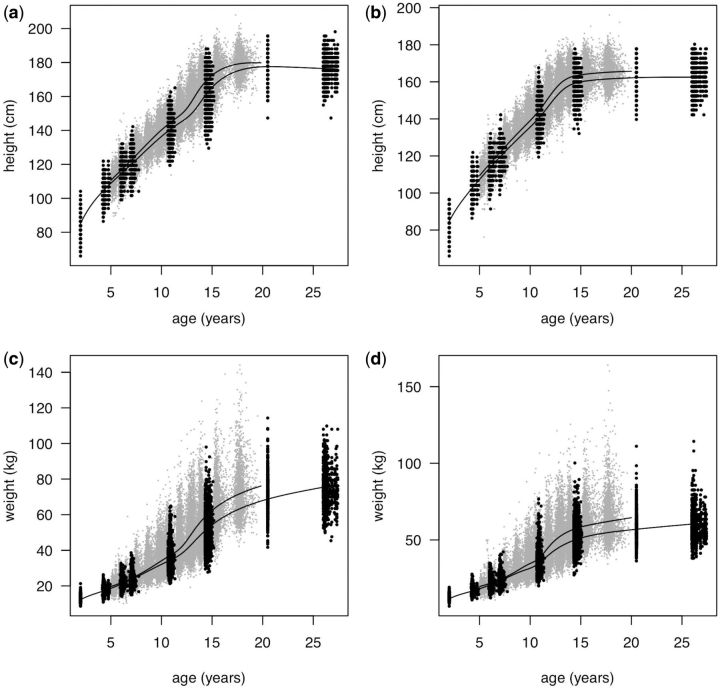
Raw data (height above, weight below, boys left, girls right) for NSHD (black) and ALSPAC (grey), and jointly fitted SITAR growth curves for the two cohorts (ALSPAC ending at age 20 years and NSHD at age 26 years).

Cleaning the NSHD height and weight data for outliers excluded 331 (1.1%) of 30 335 measurement occasions from age 2 to 26 years. The remaining 30 004 measurements, for 2574 boys (15 652 measurements) and 2327 girls (14 352 measurements), were fitted to SITAR models with 5 degrees of freedom. The analyses were then repeated with the NSHD data augmented with ALSPAC data for 5499 boys and 5397 girls, with respectively 36 560 and 37 560 measurements. The inclusion of ALSPAC allowed 6 degrees of freedom for the mean curves in the two height models. Figures 1 and 2 show the fitted growth curves.

Figure 1 compares the NSHD mean curves for height and weight by sex, estimated alone (sole—dashed lines) and with ALSPAC (joint—solid lines). The joint curves are generally smoother than the sole curves, with fewer bumps, and the sole and joint height curves are very similar in shape, though the joint curve for boys dips slightly after age 20 years. However, the weight curves differ materially after age 10 years, with the sole curves steeper before age 15 years and shallower after. This implies a real difference in the growth pattern between the two cohorts during and after puberty, even after the SITAR adjustment, with greater weight gain in ALSPAC.

Figure 2 shows the height and weight data by sex for ALSPAC (grey) and NSHD (black), illustrating the sparsity of the NSHD data during puberty. Note that, in two of the NSHD sweeps, the measurement ages were not recorded, and ages 2 and 20.5 years were imputed. Figure 2 also shows the mean curves for each cohort separately, as estimated with the joint model, where ALSPAC is above and to the left in each case (and stops at age 20 years). These differences make clear that, in the 45 years between 1946 (NSHD) and 1991 (ALSPAC), there was a clear secular trend to greater height, greater weight and earlier puberty. In particular, weight in both sexes was greater for ALSPAC at age 19 years than for NSHD at age 26 years.

### Cohort comparisons of SITAR parameters

The differences in mean curve shape between ALSPAC and NSHD shown in Figure 2 arise from differences in the underlying SITAR size, tempo and velocity parameters, estimated as fixed effects comparing the two cohorts. Table 1 summarizes these fixed effects, confirming that, in general, ALSPAC children were on average taller, heavier, earlier into puberty and faster-growing than NSHD children. The apparent exception is for girls’ weight, where the tempo effect (corresponding to age at peak weight velocity) was slightly later for ALSPAC.

**Table 1.** Mean cohort differences in SITAR growth parameters, ALSPAC relative to NSHD (coefficient and 95% confidence interval)

	Height (cm)		Weight (kg)	
Male	Female	Male	Female
Size (cm, kg)	2.3 (1.9 to 2.6)	3.4 (3 to 3.7)	3.4 (3.1 to 3.7)	4.5 (4.1 to 4.8)
Tempo (years)	–0.40 (–0.44 to –0.37)	–0.12 (–0.16 to –0.08)	–0.25 (–0.31 to –0.19)	0.14 (0.08 to 0.20)
Velocity (%)	6.8 (6.0 to 7.5)	6.5 (5.8 to 7.3)	28 (26 to 30)	36 (34 to 38)

The velocity effect was dramatic, with growth in ALSPAC 7% faster for height and no less than 30% faster for weight. It reflects SITAR's attempt to reconcile materially different curve shapes in the two cohorts. The weight increase in ALSPAC compared with NSHD was relatively greater during and after puberty than before, and to model this SITAR had to shrink the age scale (i.e. increase velocity) but also postpone puberty in ALSPAC. Thus, the tempo effects probably underestimate the true differences in puberty timing.

Table 2 shows the fit of the NSHD models, in terms of the residual standard deviations (RSDs) and the SDs of the SITAR parameter random effects, without and with the ALSPAC data, sexes pooled (*n* = 4901). In the joint models, the RSD for height was 1.6 times larger in NSHD than in ALSPAC for both sexes, whereas for weight it was 1.1 times larger. Compared with height, the tempo SD for weight was larger and the velocity SD three to four times larger in NSHD. The results for the sole and joint models in Table 2 are broadly similar, showing that adding ALSPAC did not have a dramatic effect on fit. It slightly reduced the RSD for height, but increased it for weight.

**Table 2.** Comparisons between SITAR growth parameters in NSHD derived without (sole) and with (joint) ALSPAC data, sexes combined

	Height (cm)		Weight (kg)	
Sole	Joint	Sole	Joint
Residual SD (cm, kg)	2.6	2.5	2.2	2.6
SD of size (cm, kg)	5.6	5.7	4.4	4.2
SD of tempo (%)	5.3	5.5	12.4	8.5
SD of tempo (years)	0.44	0.46	1.03	0.71
SD of velocity (%)	8.3	9.0	34	26

Correlating the SITAR random effects in the sole models with those in the joint models, all but one of the six correlations exceeded 0.94, so the addition of ALSPAC had little effect. However, for weight tempo, the correlation was much lower at 0.68, showing that adding ALSPAC affected weight tempo quite substantially. Adding ALSPAC also increased the correlations between height tempo and weight tempo, from 0.45 to 0.60 in boys and from 0.24 to 0.56 in girls, implying improved estimates, since the two are known to be strongly correlated.^16^

The random effects for size and velocity in each model were very strongly correlated with each other (sole models: 0.85 for height, 0.99 for weight; joint models: 0.76 height and 0.97 weight). These are much larger than found in a previous SITAR analysis, and probably reflect the instability of modelling NSHD with sparse data.^7^ In contrast, the correlations of tempo with size and velocity were all <0.2 for height, and for weight in the joint model, but for weight in the sole model they were both much higher at ∼0.7. So adding ALSPAC enabled the weight-tempo effects to be separated from weight size and velocity. This latter contrast probably best explains the differences between the sole and joint models.

### Comparison of SITAR tempo with puberty timing

Table 3 shows the correlations in the NSHD boys and girls between the measures of puberty timing and the SITAR parameters from the sole NSHD and joint NSHD–ALSPAC models. As expected, puberty timing correlated positively with SITAR tempo, with correlations of around 0.5 (*P* < 0.0001). The addition of ALSPAC consistently increased each correlation, only weakly for height but dramatically so for weight, where the correlation doubled, though it was still less than for height. Thus, despite the apparently small impact on the random effects of including ALSPAC, it appreciably improved the estimate of puberty timing, particularly for weight. By contrast, the correlations of puberty timing with SITAR size and velocity were weak, particularly with the joint models, as would be expected.

**Table 3.** Correlations of NSHD SITAR parameters with NSHD puberty timing based on menarche questionnaire (girls) and school doctor examination (boys), as obtained from the sole NSHD models and joint NSHD–ALSPAC models

	Male		Female	
Sole	Joint	Sole	Joint
Height	Size	–0.10	–0.11	0.05	0.06
	Tempo	0.48	0.55	0.58	0.58
	Velocity	–0.25	–0.20	0.03	–0.02
Weight	Size	–0.08	–0.05	–0.13	–0.11
	Tempo	0.24	0.45	0.18	0.46
	Velocity	–0.09	–0.04	–0.13	–0.11

### Relation between SITAR growth parameters and bone outcomes

The influence of pubertal growth on later bone outcomes is explored in Table 4, where the outcomes are regressed on the SITAR height and weight parameters, adjusted for sex, and in addition for height and weight at the time of the scan. The analysis is restricted to 1321 subjects (642 men, 679 women) with complete data on SITAR parameters, bone outcomes and anthropometry at age 60–64 years.

**Table 4.** Significance (*t*-values) of regression coefficients of NSHD bone outcomes on SITAR parameters, without and with body size adjustment, sexes combined (*n* = 2321)

Bone outcome	Trabecular vBMD		Diaphysis CSA	
Model	1^a^	2^b^	1	2
Height size	**–2.4**	–1.3	**2.6**	–1.6
Height tempo	**–3.4**	**–3.6**	–0.2	–0.1
Height velocity	1.0	0.8	1.3	1.2
Weight size	–0.1	0.0	**5.2**	**5.2**
Weight tempo	–0.5	–0.1	–2.2	**–2.5**
Weight velocity	0.5	–0.3	**–2.7**	**–2.9**
Female sex	–15	–4.1	–47	–11
Height @ 60–64		–0.4		4.0
Weight @ 60–64		4.6		2.9
Adjusted *R*^2^ (%)	15.9	17.1	65.8	66.4
Growth impact (%)^c^	17.5	17.9	25.8	15.3

^a^ Model 1: SITAR height and weight parameters plus sex.

^b^ Model 2: as Model 1 plus height and weight at age 60–64 years.

^c^ Impact on outcome attributable to growth pattern—see text.

Bone outcomes, height and weight are all log-transformed. SITAR parameter coefficients significant at *P* < 0.02 are shown in bold.

In summary, the pattern of pubertal growth has a material impact on later bone health, with or without later body size adjustment. However, in detail, the relationships between pubertal growth and bone health vary by outcome.

For trabecular vBMD, height tempo is very strongly associated (*P* < 0.001), its negative sign indicating that earlier puberty is associated with greater vBMD. Model 1 explains 16% of the variance, and the difference in predicted outcome for the extremes of growth represented by the model (i.e. +2 vs –2 SDs of the linear predictor—see the ‘Methods’ section) is a substantial 17.5% (Table 4). Omitting the other SITAR parameters from the model reduces the impact attributable to growth to 15% (not shown), so that vBMD is 15% less in those with late as opposed to early puberty, adjusted for sex. [This 15% figure corresponds to 4 SDs of height tempo (Table 2) times its regression coefficient of –0.65 SE 11.] Adjusting for current body size (Model 2) increases the variance explained to 17% but hardly alters the growth impact, whereas the growth impact attributable to height tempo alone (refitting the model omitting the other SITAR variables) is a still impressive at 13%. The SD of vBMD is 26%, so this corresponds to a half SD effect size.

Conversely, for diaphysis CSA, the important SITAR parameters are weight size (*P* = 0.0002), weight velocity (*P* = 0.008), height size (*P* = 0.01) and weight tempo (*P* = 0.05), together indicating a strong effect of pubertal body size (Table 4, Model 1). The outcome is sexually dimorphic, as shown by the high *t* value for sex which accounts for 56% of the variance on its own, and 64% including current body size (not shown). The impact of growth on the outcome is 26%, falling to 15% adjusted for current body size (Model 2). The negative coefficients for weight tempo and weight velocity mean that greater weight, and to a lesser extent earlier puberty and slower growth velocity, are positively associated with CSA. Thus, pubertal weight growth and current body size have separate and independent effects, the former amounting to three-quarters of an SD of CSA (which is 21%).

## Discussion

### Statement of findings

The study shows that pubertal growth in NSHD can be summarized compactly in terms of subject-specific SITAR parameters, and that the addition of ALSPAC data to the model provides smoother mean curves and more robust estimates of the weight-tempo random effects in NSHD. The resulting SITAR parameters are strongly associated with bone health outcomes measured 50 years later; vBMD is 13% less in late maturers compared with early, and 15% of the variance in diaphysis CSA is explained by the pattern of pubertal weight gain. The growth pattern corresponding to later bone health is summarized by greater weight size, earlier weight tempo and lower weight velocity (Table 4).

As a subsidiary finding, the mean differences in the SITAR parameters between NSHD and ALSPAC (see Table 1) reflect marked upward secular trends in height and weight, in terms of both size and velocity, and earlier puberty for boys but less so for girls. Indeed mean weight tempo for girls was seven weeks later in ALSPAC. However, the mean curves in Figure 2 show that puberty was consistently earlier in ALSPAC in both sexes, indicating that the large velocity effect swamped the tempo effect.

The greater robustness of the joint weight-tempo estimates is demonstrated in two ways: they are more highly correlated with height tempo (*r* ∼ 0.6) and they correlate more highly with contemporaneous questionnaire responses to puberty timing. Some example R code is provided as a Supplementary Data file.

In detail, the modelling is imperfect. The height RSDs of ∼2.5 cm are appreciably larger than for two other SITAR published models,^7^ where both RSDs were <0.8 cm. However, these other studies started at age 9 years whereas NSHD started at age 2 years, and this will have inflated the error. Looking at the SDs of the random effects, those for height are all smaller than in previously published studies (∼6 cm for size, ∼1 year for tempo and ∼20% for velocity^7^^,^^8^). In addition, the NSHD mean curves, though improved when estimated jointly with ALSPAC, are still lumpy (see Figure 1), and the weight curves are forced to be steeper after puberty when ALSPAC is added. This is because pubertal weight gain is greater in ALSPAC than in NSHD, and it violates the SITAR assumption that the curves can be made the same shape by suitable choice of SITAR parameters. However, this does not apply to the height curves, where the assumption holds and the curves for the two cohorts are essentially the same after SITAR adjustment.

An important design question when piggy-backing data, as done here with ALSPAC, is getting the right mix of original and supporting data—if the added data are too few, they make no difference (as was found by initial experimentation with a much smaller growth study than ALSPAC^8^), whereas, if they are too many, they may swamp the original data. It was important here to allow the RSDs to differ for the two datasets, showing that height was more noisy in NSHD than in ALSPAC. This may be due partly to the missing age of measurement at age 2 years.

The results of the regression models relating bone outcome at age 60–64 years to pubertal growth as summarized by SITAR demonstrate the complexity both of pubertal growth and its relationship with bone status 50 years later. Trabecular vBMD relates to age at peak height velocity,^11^ whereas diaphysis CSA is affected by all three facets of pubertal weight growth. These findings illustrate the extra information derived from the SITAR model, with its tempo, size and velocity parameters, over and above reported pubertal timing, even in studies like NSHD where the reports are prospectively obtained and clinically assessed.^11^ Each parameter represents a different aspect of growth. For a bone to be ‘fit-for-purpose’, it must appropriately adapt to longitudinal growth^11^ and to the loading it undergoes, which will change with the timing, speed and mean weight gain, and it is consistent with the findings for CSA. For vBMD and CSA, the risk attributable to pubertal growth, i.e. the difference in predicted outcome for those 2 SDs below as opposed to 2 SDs above the mean of the linear predictor based on the SITAR parameters, is substantial and clinically important, at around 15% adjusted for current body size, which would translate to a clinically relevant increased risk of fracture.^11^^,^^17^^,^^18^ Given that a 1-SD reduction in BMD^17^ results in a doubling of fracture risk, the differences in the current study represent a 1.5- to 2-fold increased risk of fracture between the two extremes.

### Strengths and limitations

Strengths of the study are the large sample size, the long period of follow-up and well-characterized bone outcomes in NSHD,^11^ plus the large number of height and weight measurements from ALSPAC through puberty. The use of pQCT enables the investigation of multiple aspects of bone strength, which no other bone densitometry technique can provide. Thus, it has been possible to understand how the different aspects of growth may influence skeletal health in later life.

A limitation is that SITAR assumes the same underlying growth curve for all individuals, after adjusting for size, tempo and velocity differences, yet the mean curves for weight in NSHD and ALSPAC are systematically different, with weight rising faster through puberty in ALSPAC than in NSHD. This affects the shape of the mean curve, and probably the velocity random effects as well, but the size and tempo random effects are likely to be relatively robust.

### Conclusions

In conclusion, individual patterns of pubertal growth in NSHD, summarized by subject-specific SITAR parameters, are better estimated when the relatively sparse NSHD data are augmented with more frequent ALSPAC data. Relating the SITAR parameters to bone outcomes in later life shows that pubertal growth, particularly earlier puberty, greater weight and slower weight gain, has a long-lasting effect on bone health, which is to a large extent independent of contemporary body size.

## Funding

This work was supported by the UK Medical Research Council (MC_UU_12019/1 and MC_UU_12019/4 for NSHD, DK, SM and RH, MC_UU_12013/5 and MC_UU_12013/9 for IEU, MR/M020894/1 for LDH, MR/M012069/1 for TJC, MC_UP_1005/1 for WJ, U105960371 for KW, MC_UU_12015/2 for KO), the Wellcome Trust (092731) and the University of Bristol for ALSPAC.

## Supplementary Material

Supplementary Data

Supplementary Data
