# Supplementary material for: Using Super-Imposition by Translation And Rotation (SITAR) to relate pubertal growth to bone health in later life: the Medical Research Council (MRC) National Survey of Health and Development
Source: Int J Epidemiol. 2016 Jul 27;45(4):1125–34. doi: 10.1093/ije/dyw134 (PMC5841778; doi:10.1093/ije/dyw134)
Supplement: Supplementary Data [file supp_45_4_1125__index.html]

Using Super-Imposition by Translation And Rotation (SITAR) to relate pubertal growth to bone health in later life: the Medical Research Council (MRC) National Survey of Health and Development — Supplementary Data 

# Using Super-Imposition by Translation And Rotation (SITAR) to relate pubertal growth to bone health in later life: the Medical Research Council (MRC) National Survey of Health and Development

## Supplementary Data

files

- Supplementary Data - txt file
